# New Mode of Treating Ruptures of the Urethra

**Published:** 1830-10-01

**Authors:** 


### MISCELLANIES.

### LII.

### New Mode of treating Ruptures of the Urethra.

M. Desruelles, surgeon to the Val de Grace, has published in the Journal des Progrés, No. XVIII, a kind of memoir on lacerations of the urethra. Several cases of rupture of this canal and considerable hæmorrhage in consequence, are related. In one, there being inflammation of the urethra and retention of urine, the employment of the catheter gave rise to such formidable hæmorrhage that the patient was lost. In another the individual was nearly destroyed by bleeding, in consequence of the introduction of the caustic bougie. In a third case acute inflammation of the urethra was followed by rupture, infiltration of urine into the surrounding parts, and death. M. Desruelles remarks that horse exercise is one of the most frequent causes of this accident in persons labouring under chronic inflammation of the urethra, for he has almost always observed it in horsemen. As laceration of the urethra is generally combined with, or dependent on contraction of the canal, M. Desruelles conceives that it is a matter of importance to dilate the strictured part, without interfering with the remainder of the urethra. He has therefore employed a hollow silver tube, one or two inches in length, and of different diameters, which is introduced into the contracted part, and withdrawn by means of a silken thread attached to one of its extremities. Before its introduction it is necessary to introduce a bougie, in order to learn the requisite size of the tube, and the depth to which it must be passed. The silken thread is then passed through what may be termed a conductor, the tube being thus attached to its extremity. The tube is then pushed before the conductor into the strictured part of the urethra, where it is left, while the conductor is withdrawn. The silken thread being fastened round the penis, or to proper tapes, prevents the tube from slipping farther into the urethra or becoming impacted there. The tube is left in for twenty-four or thirty-six hours. We cannot say we see any great advantage in this complicated manœuvring. It is much more clumsy, and much more difficult than the introduction of a silver catheter, more likely to create irritation, and not a whit more adapted to effect a cure. In the abscess of the perineum, depending on fistulous opening into the urethra and stricture, such a mode of proceeding would in most cases be impracticable, for it is often impossible, always difficult to introduce the more manageable bougie. Besides, if any accident happened to the silk thread, or if severe inflammation of the urethra and surrounding cellular membrane supervened, it would be but an awkward thing at the best to have a little tube pent up in the inmost recesses of the urethra. However, our readers may form their own opinions on the subject.

---

